# The exacerbated hypokalemia in membranous glomerulonephritis due to proximal tubular injury: a neglect issue from a case report and literature review

**DOI:** 10.1186/s12882-023-03130-4

**Published:** 2023-04-15

**Authors:** Chih-Hao Chang, Hui-Jung Yu, Yi-Chou Hou

**Affiliations:** 1 Department of Pulmonary and Critical Care Medicine, Department of Internal Medicine, Municipal Tucheng Hospital, New Taipei City, Taiwan; 2 Department of Pathology, Cardinal Tien Hospital, School of Medicine, Fu-Jen Catholic Univer, sity, New Taipei City, Taiwan; 3 Department of Internal Medicine, Cardinal Tien Hospital An-Kang Branch, School of Medicine, Fu-Jen Catholic University, Hsin-Tien District, New Taipei City, 23155 Taiwan

**Keywords:** Nephrotic syndrome, Membranous glomerulonephritis, Hypokalemia

## Abstract

**Background:**

Membranous glomerulonephritis is the most common primary etiology for the nephrotic syndrome in adults. Beyond the clinical hallmark of nephrotic syndrome such as fluid overloading, dyslipidemia and hypoalbuminemia, the dysregulated homeostasis of potassium and its possible mechanism is seldomly discussed, and its association with the clinical course of membranous GN is lacking.

**Case presentation:**

A 65 year-old female attended to our emergent department for progressive lower leg edema after taking 15-h of flight. Hypoalbuminemia and hyperlipidemia were both noted, and 24-h urinary total protein was up to 17,950 mg/day. Elevated creatin-phospho-kinase developed at the initial presentation with hypokalemia due to excressive renal excretion. Glycosuria without elevated glycated Hemoglobin occurred. The pathology of kidney biopsy revealed subepithelial immunocomplex deposits with spike formation in the electron microscopy and the positive anti-Phosphlipase A2 receptor antibodies(PLA-2R) with hallmark of membranous glomerulonephritis. In the light microscopy, the vacuolization of proximal tubules was noted, which contributed to the potassium wasting. After 1 year following up duration, the patient’s proteinuria persisted after maintenance treatment with calcineurin inhibitor.

**Conclusion:**

Hypokalemia is a neglected issue in the membranous glomerulonephritis. Unlike the previous literature, our patient had the vacuolization of proximal tubule at the initial presentation with hypokalemia, which might contribute the potassium wasting. The proximal tubular damage with hypokalemia might be a predictive factor for membranous glomerulonephritis.

## Introduction

Nephrotic syndrome, characterized by proteinuria, dyslipidemia, hypoalbuminemia and systemic edema is related to glomerular protein loss into urine. Although the protein loss in the urinary lumen can be absorbed from the proximal tubules, dysregulation of potassium, especially hypokalemia is not so common in patients with nephrotic syndrome. According to the previous literature, membranous glomerulonephritis associated with Liddle’s-like syndrome is noted, and the possible mechanism had been discussed. We would like to present a female with initial presentation of massive proteinuria with reversible hypokalemia, which was confirmed with membranous glomerulonephritis and proximal tubular vacuolization. In this case, the detailed clinical significance with hypokalemia, proximal tubular injury and clinical relevance would also be aroused.

## Case presentation

**Fig. 1:**
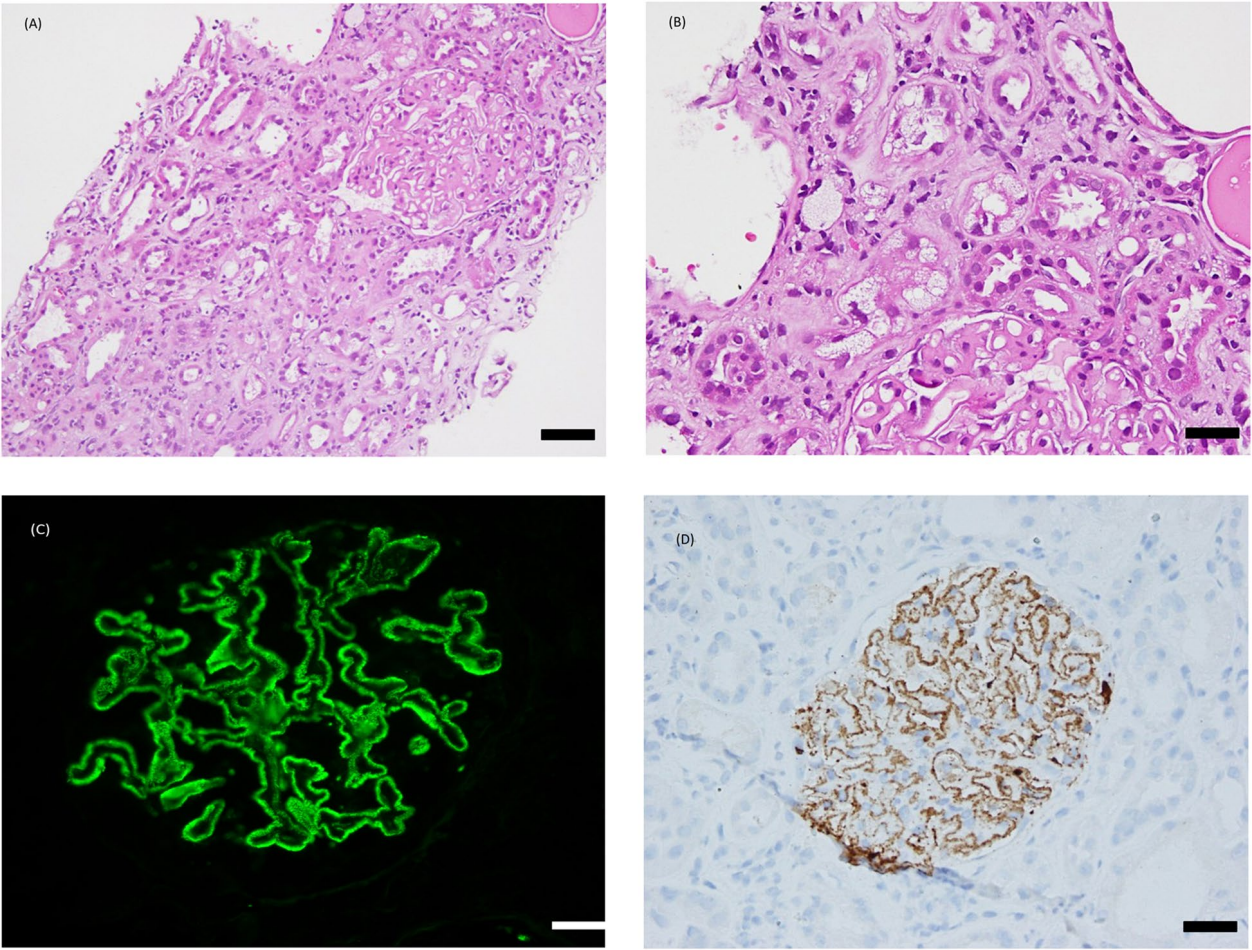
**a** and **b** Light microscopy demonstrated diffuse membranous thickening within the glomerulus with abundant optically clear intracytoplasmic vacuoles, which ranged from predominantly large and coarse to fine, involving both the proximal tubular epithelial cells. The vacuoles appeared empty with all routine stains (including hematoxylin and eosin. Hematoxylin and eosin, original magnification × 40 for panel (**a**) and × 100 for panel (**b**). Scale bars 100 μm (**a**) and 20 μm (**b**). **c** Immunoflorescencent stain revealed diffuse global granular capillary loop staining for IgG. Scale bars 20 μm. The immunofluorescence stain was illustrated by Leica DM2500, Germany (**d**) Immunohistochemical stain revealed granular staining along basement membrane for PLA2R. Scale bars 20 μm. The light microscopy and immunohistochemical stain were illustrated by Nikon E600, Japan

**Fig. 2:**
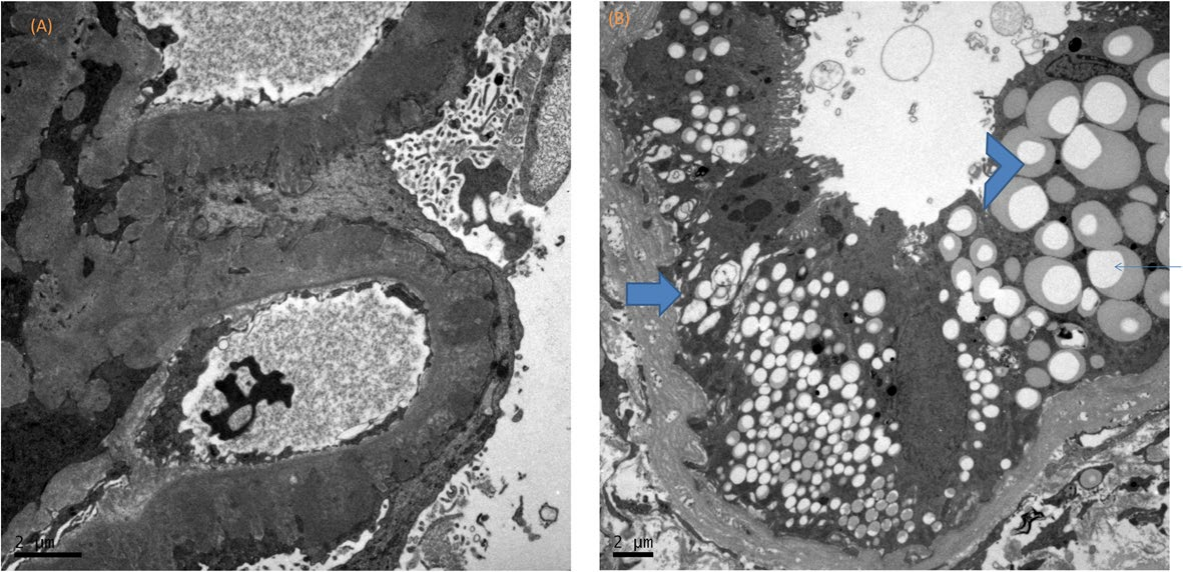
**a** Electron microscopy demonstrated the subepithelial immune complex deposition with formation of perpendicular spike similar to glomerular membrane (illustrated by Hitachi S-3000N, Japan. The visceral podocyte foot process effacement was also demonstrated. **b** The electron microscopy demonstrated intracytoplasmic electron lucent vacuoles (arrow) of various sizes in the proximal tubular epithelial cells. Beyond the electron-lucent vacuoles, the vacuoles with lipid droplet (arrowhead, with crescent-like content) also presented within the vacuoles. Original magnifications × 3000. Scale bar 2 μm

**Fig. 3:**
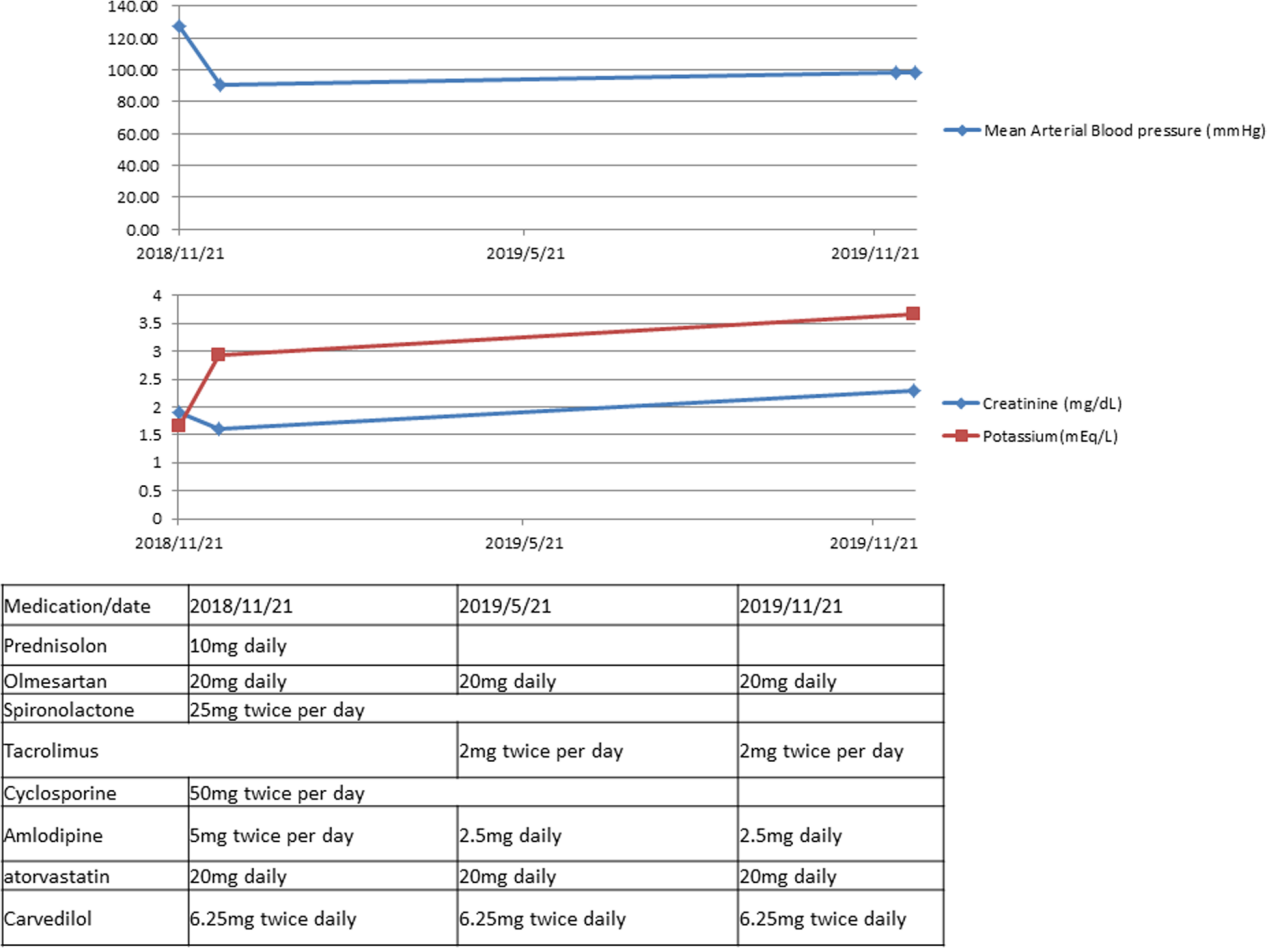
The variations of serum creatinine, potassium, mean arterial blood pressure and the medication

A 65 year-old female attended to our emergent department for progressive lower leg edema after taking 15-h of flight. The patient had been diagnosed with hypertension and she was under clinics following-up in Canada. No previous diuretics or other antihypertensive medication exposure was noted before this episode although hypertensive history was told. According to the patient’s clinician, no previous serum potassium disorder was documented because the patient did not take routine examination. On 2018 11/15, bilateral lower leg edema developed after she landed off in Taiwan and then persisted for 2 days. Beyond the edema, the patient denied other associated symptoms such as shortness of breath, chest pain, decrease in urine output, fever, nausea, vomiting, bloody or tarry stool or palpable nodule within breast or neck, etc. No other exposure history to drug or substance was mentioned. Therefore, the patient was referred to emergent department. High blood pressure (185/97 mmHg) with normal sinus rhythm was noted at ER. Table 1 illustrated the serial laboratory change. The laboratory data revealed profund hypokalemia (Potassium: 1.66 mEq/L). and elevated serum blood urea nitrogen and creatinine (27 mg/dL/1.90 mg/dL). Elevated creatin-phospho-kinase (7683U/L) was noted without associated elevation of glutamic oxaloacetic transaminase or glutamic pyruvic transaminase were also occurred at the same time. The urine analysis demonstrated the positive glucose (3 +), urine protein (4 +), urine RBC without dysmorphic change (3 +), and negative for cast (not found). The blood gas analysis didn’t demonstrate acidosis or alkalosis. Hypoalbuminemia and hyperlipidemia were both noted, and 24-h urinary total protein was up to 17,950 mg/day. Under the impression of nephrotic syndrome with hypokalemia, the patient was admitted. TTKG was 12. 8, and the serum levels of autoimmune disease (including Anti-nuclear antibody, C3, C4), aldosterone, plasma renin activity, Hepatitis B surface antigen, anti-hepatitis C antibody, paraprotein, were negative. No hyper-or hypothyroidism was noted. Serum magnesium was within normal limit. The patient’s hypokalemia was refractory to intravenous potassium supplement with dosage of 140 mEq/day since 2018/11/22. Abdominal computer tomography was performed and there was no adrenal lesion. After spironolactone 25 mg twice daily use, valsartan 80 mg once daily use were given, the serum potassium was stabilized (K: 3.1 mEq/L). Kidney biopsy was performed due to nephrotic syndrome. Figure 1 illustrated the diffuse membranous thickening within the glomerulus with abundant optically clear intracytoplasmic vacuoles, which ranged from predominantly large and coarse to fine, involving both the proximal tubular epithelial cells under light microscopy demonstrated. No crescents, fibrins or necrosis within glomerular Bowman’s space was noted. Large irregular-sized, coarse vacuoles in the cytoplasm of tubular epithelial cells were noted. 20% of interstitial fibrosis and interstitial lymphocytic infiltrates was noted. Immunohistochemical stain revealed positive stain for PLA2-R receptor antibody and IgG4. The immunofluorescence microscopy demonstrated diffuse global granular capillary loop staining for IgG (3 +), C3 (2 +), C4 (1 +), C1q (1 +). Figure 2 illustrated the eletromicroscopy. The electron microscopy demonstrated subepithelial immunocomplex deposits with spike formation. Visceral foot process effacement was noted. Lipid droplets and lysosomal vacuoles in tubular epithelial cells were also noted. Intracytoplasmic electron lucent vacuoles (arrow) of various sizes in the proximal tubular epithelial cells were found. Besides, the vacuoles with lipid droplet (arrowhead, with crescent-like content) also presented within the vacuoles. Based on the pathologic finding, membranous glomerulonephritis was confirmed. Renal wasting hypokalemia was also confirmed. Figure 3 illustrated the patient’s maintenance medication along with the variation of serum creatinine, potassium and mean arterial blood pressure. The patient received tacrolimus 2 mg twice daily, amlodipine 2.5 mg once daily, olmesartan 20 mg once daily, atorvastatin 20 mg once daily, carvedilol 6.25 mg twice daily as maintenance treatment. In 2019/12, the patient’s serum BUN/Cr was 30/mg/dL/2.3 mg/dL. The serum potassium level was 3.60 mEq/L. The daily urinary total protein excetion was 14,337.54 mg/g. In 2022/2, the patient started to receive maintenance hemodialysis.

**Table 1:** The serial variation of the biochemical, serologic and urinary results of the patient

	2018/11/21	2018/11/22	2018/11/24	2018/11/25	2018/11/27	2018/12/12	2019/12/23
White blood cell count ( /μL)	11800				6100	13460	
Hemoglobin(g/dL)	12.1				8.3	9.5	10.4
Platelet count (/μL)	359000				200,000	535000	
Eosinophil (%)	0.5				3.8		
Blood urea nitrogen (mg/dL)	27			14		27	30
Creatinine (mg/dL)	1.9	1.43		1.19	1.55	1.62	2.3
Sodium (mEq/L)	138					142	144
Potassium (mEq/L)	1.66	1.57	3.04	3.63	3.1	3.15	3.67
Calcium(mg/dL)	8.1						7.9
Phosphate(mg/dL)			2.73				
Uric acid (mg/dL)			4.0				
Creatine phosphokinase (U/L)	7683	4450	8840	5514	943	64	
Troponin I ( ng/mL)	0.053						
Blood osmolarity(mmol/L)	295						
pH	7.43						7.33
pCO2 (mmHg)	33.2						35.9
HCO3(mmol/L)	22.2						18.6
Magnesium (mg/dL)	2						
Albumin(g/dL)		2.65					2.73
Triglyceride		343					288
HbA1c (%)		5.8					
Low Density lipoprotein(mg/dL)		174					137
Cholesterol		312					253
Urine Potassium (mEq/L)	22.32						
Urine osmolaity (mmol/L)	267						
TTKG	12.8						
Urine glucose		3 +					3 +
Urine pH		7.5					7.0
24 h total protein(mg/24 h)			17950			14463.83	14337.54

*HCO3*- Bicarbonate, *pCO2* carbon dioxide, *TTKG* The Transtubular Potassium Gradient

## Discussion

Primary membranous glomerulonephritis is the most common entity contributing to the nephrotic syndrome in the adults. Subepithelial immune complex deposition induces the foot process effacement in the podocyte and therefore increase the glomerular protein filtration into the urinary space. Physiologically, the proximal tubules reabsorb the amino acid and filtered protein. In the nephrotic syndrome, the elevated total cholesterol and triglyceride parallelly occur with the significant increase in apoA-I, apoA-IV, apoB, apoC, and apoE levels and the apoC-III to apoC-II ratio. Such alternation of lipoprotein induce the abeit of hepatic lipase activity, deficiencies of lipoprotein lipase, very low density lipoprotein receptor and upregulation of angiopoietin-like protein 4 and decrease the lipid metabolism within the skeletal muscle and fat tissue. Therefore, the increased lipid influx and the fatty acid oxidation not only poses damage on the glomeruli but also the tubulointerstitium. Among the renal tubules, proximal tubule is the most vunerable area for the lipid mediated damage in nephrotic syndrome. From the renal biopsy, lipid droplet was noted in the proximal tubular epithelial cells, and the proximal tubular cell vacuolization was documented, which was compatible to the nephrotic syndrome mediated acute tubular necrosis.

In this patient, profund hypokalemia was noted at the initial presentation. High TTKG demonstrated that the renal wasting of potassium contributed to her hypokalemia. In the initial phase, the transcellular shifting should be excluded. It has been known that the transcellular shifting of potassium is initiated by the thyroid hormone, catecholamine, excessive aldosterone, insulin function or alkalosis [1, 2]. We checked the serum thyroid function, aldosterone, PRA and serum pH, which was within the normal range. Although the transient increase of catecholamine or insulin was not measured immediately, the transcellular shifiting is less likely. Hypertension was document initially with neutral pH in the serum. The differential diagnosis of hypokalemia should be established initially based on the blood pressure and acid–base status. In the hypertensive status, plasma renin activity and aldosterone provides further diagnostic value. In this patient, low aldosterone and PRA arouse the possibility of congenital adrenal hyperplasia, Cushing’s syndrome and minerocorticoid ingestion/Liddle syndrome. Besides, the anti-hypertensive medication within 2 weeks of measurement could influence the serum concentration of PRA and aldosterone. However, the negative exposure history of substance or alternative medicine excluded the evidence. Besides, the patient’s acid–base status was not alkalotic. When tracing the algorithm for hypokalemia, no definite diagnosis might be confirmed [3]. Hypokalemia nephropathy, which is clinically diagnosed with hypokalemia, metabolic alkalosis, polyuria with progressive loss of glomerular filtration might also contribute to the hypokalemia in this patient. However, the clinical course and the pathologic finding might not fully compatible with the diagnosis. First, the patient’s hypokalemia developed immediately after the rhabdomyolysis occurred and then subsided after treatment. Hypokalemia contributed to the development of rhabdomyolysis. Since the pre-admission serum potassium concentration was not available, the sequence between hypokalemia and rhabdomyolysis might not be distinguished. Second, the pathologic characteristics were not fully compatible with the hypokalemia nephropathy. The proximal tubular vacuolization and the interstitial lymphocytic infiltration was noted in the kidney biopsy in this patient. However, the renal tubular hypertrophy was not documented in the medullary collecting duct or the cortical collecting ducts. The immobile status during the airflight trip worsened the myoglobulin released and therefore worsened the proximal tubular injury, which might develop the transient glycosuria without elevated plasma glycosylated hemoglobin (5.8%). However, the development of proximal tubular damage and the clinical course of membranous GN was not elucidated [4]. Furthermore, the development of proximal vacuolization might be the early manifestation of tubular injury [5, 6]. The proximal tubular injury might be the early manifestation of further tubulointerstitial fibrosis. Our patient received maintenance hemodialysis in 2022/2. From this aspect, we hypothesized that the proximal tubular vacuolization might be a possible predictive marker for clinical course. To validate the hypothesis, it is essential to quantify the extent of the proximal tubular vacuolization in patients with nephrotic syndrome [7]. Larger extent of sample size might be provide the relevant evidence.

The association between nephrotic syndrome and hypokalemia was also discussed to elucidate the molecular pathogenesis and its clinical relevance. The association between hypokalemia and nephrotic syndrome has been discussed since 1980’s [8]. Yamaguichi demonstrated a case of membranous GN patient with the hypokalemic presentation, and Liddle’s syndrome-like pattern, which had hypertension with or without metabolic alkalosis (Pagani L, et al., Hypertension. 2018, 2: 273–279) [9]. From the aspect of distal tubular damage, the albuminuria alone might worsen the distal potassium excretion by renal outer medullary potassium channel (ROMK) [10]. From the report based on Perssen, the urinary plasmin filtered from plasminogen could influence the γ-subunit of epithelial sodium channels [11]. The activated ENaC could enhance the reabsorption of the sodium and therefore excrete the potassium vial renal outer medullary potassium channel. The increased absorption of sodium further suppressed the serum renin and aldosterone secretion because of volume expansion. As the patient demonstrated by Yamaguichi et al., the hypokalemia could be corrected by the supplement of olmesartan and the spironolactone. For the treatment of hypokalemia in proximal tubule dysfunction such as Fanconi syndrome, the potassium supplement along with the sodium bicarbonate and fluid repletion is cornerstone [12]. However, the suppressed renin and aldosterone activity due to the fluid overloading status in nephrotic syndrome might hamper the standard management, and therefore the hypokalemia might be corrected by spironolactone even with the proximal tubular dysfunction. The potassium wasting mediated by the proximal tubular injury might be the major and a neglect issue when managing the membranous glomerulonephritis. The filtered fatty acid bearing albumin could contribute to proximal tubular injury and sequential tubulointerstitial fibrosis by recruiting macrophage [13]. The proximal tubular vacuolization has been noticed in the calcineurin-induced toxicity and the diabetes mellitus subjects. The vacuolization reflected the glycogen storage [14], and it might be associated with excessive oxidative stress and fibrogenic cytokine such as transforming growth factor β1 [15]. In this patient, massive proteinuria and declining glomerular filtration persisted even after treatment. To answer the sequence between the proximal dysfunction, nephrotic syndrome and hypokalemia, sequential kidney biopsy after the diagnosis confirmed might provide further understanding.

## Data Availability

Records and data pertaining to this case are in the patient’s secure medical records in Cardinal Tien Hospital. The relevant material could be provided by corresponding author for reasonable request.

## Abbreviations

PLA2-R: Anti-Phosphlipase A2 receptor Antibodies
TTKG: The Transtubular Potassium Gradient
